# Combining Heart Rate Variability and Oximetry to Improve Apneic Event Screening in Non-Desaturating Patients

**DOI:** 10.3390/s23094267

**Published:** 2023-04-25

**Authors:** Sofía Martín-González, Antonio G. Ravelo-García, Juan L. Navarro-Mesa, Eduardo Hernández-Pérez

**Affiliations:** 1 Institute for Technological Development and Innovation in Communications, Universidad de Las Palmas de Gran Canaria, 35017 Las Palmas de Gran Canaria, Spain; 2 Interactive Technologies Institute (ITI/LARSyS and ARDITI), 9020-105 Funchal, Portugal

**Keywords:** apnea detection, cepstrum coefficients, detrended fluctuation analysis, heart rate variability, linear and nonlinear analysis, non-desaturating patients, oxygen saturation, recurrence quantification analysis

## Abstract

In this paper, we thoroughly analyze the detection of sleep apnea events in the context of Obstructive Sleep Apnea (OSA), which is considered a public health problem because of its high prevalence and serious health implications. We especially evaluate patients who do not always show desaturations during apneic episodes (non-desaturating patients). For this purpose, we use a database (HuGCDN2014-OXI) that includes desaturating and non-desaturating patients, and we use the widely used Physionet Apnea Dataset for a meaningful comparison with prior work. Our system combines features extracted from the Heart-Rate Variability (HRV) and SpO_2_, and it explores their potential to characterize desaturating and non-desaturating events. The HRV-based features include spectral, cepstral, and nonlinear information (Detrended Fluctuation Analysis (DFA) and Recurrence Quantification Analysis (RQA)). SpO_2_-based features include temporal (variance) and spectral information. The features feed a Linear Discriminant Analysis (LDA) classifier. The goal is to evaluate the effect of using these features either individually or in combination, especially in non-desaturating patients. The main results for the detection of apneic events are: (a) Physionet success rate of 96.19%, sensitivity of 95.74% and specificity of 95.25% (Area Under Curve (AUC): 0.99); (b) HuGCDN2014-OXI of 87.32%, 83.81% and 88.55% (AUC: 0.934), respectively. The best results for the global diagnosis of OSA patients (HuGCDN2014-OXI) are: success rate of 95.74%, sensitivity of 100%, and specificity of 89.47%. We conclude that combining both features is the most accurate option, especially when there are non-desaturating patterns among the recordings under study.

## 1. Introduction

Sleep apnea is a widespread sleep respiratory disorder characterized by disrupted breathing during sleep. Sleep apnea events are classified according to the associated respiratory effort. Obstructive Sleep Apnea (OSA) occurs when the upper airway collapses and airflow is obstructed. However, when the upper airway is open and the respiratory drive is absent or inhibited, i.e., there is no respiratory effort, it is considered Central Sleep Apnea (CSA). Both types occur in Mixed Sleep Apnea (MSA). Although there are often CSA episodes in OSA patients, pure CSA is uncommon.

OSA is the most common type of sleep apnea [1]. It is considered a public health problem because of its high prevalence, which is 4% among men and 2% among women [1], as well as its serious health implications, such as cardiovascular disorders, strokes, glucose metabolism abnormalities, sudden death, or depression, that lead to increased mortality rates [2]. However, other symptoms also severely affect quality of life, including daytime drowsiness, snoring, weight gain, irritability, or memory problems [3,4]. The severity of OSA is measured by the Apnea-Hypopnea Index (AHI), which represents the mean number of breathing pauses per hour. Apnea in adults is scored when there is a drop in the peak signal excursion by ≥90% of the pre-event baseline using an oronasal thermal sensor (diagnostic study), PAP device flow (titration study), or an alternative apnea sensor, for ≥10 s. According to the definition, apneas may or may not be accompanied by desaturations, but they will always be labeled as apneas. Therefore, apneic events may be non-desaturating. Hypopnea in adults is scored when the peak signal excursions drop by ≥30% of the pre-event baseline, using nasal pressure (diagnostic study), PAP device flow (titration study), or an alternative sensor, for ≥10 s in association with either ≥3% arterial oxygen desaturation or an arousal [5]. Apneas and hypopneas have similar pathophysiology and, in general, similar impacts on patients [6]. AHI values lower than 5 are considered normal, and patients with AHIs over 5 are diagnosed with OSA and ranked according to the following ranges: (5, 15): mild sleep apnea; (15, 30): moderate sleep apnea; >30: severe sleep apnea.

### 1.1. Motivation and Problem Description

Due to the severity of OSA, early diagnosis of the disease is strongly recommended so that patients can receive appropriate and effective treatment. Polysomnography (PSG) is the gold standard diagnostic tool for OSA. It records different physiological signals throughout the night at the hospital, and it is supervised by qualified staff. The process is inconvenient for the patient, time consuming, and very expensive for the health care system. Therefore, efforts are geared towards developing alternative and simplified automatic, as well as portable, OSA detection techniques that are based on fewer physiological signals, thus reducing the time required for a final diagnosis. Several automatic OSA detection approaches have been reported in the literature [7]. Some carry out patient sound analysis [8,9,10], while others study airflow [11,12,13], abdominal and thoracic movement signals [14], and even voice analysis [15,16]. However, most include statistical pattern recognition based on characteristics extracted from single-lead electrocardiogram (ECG) signals [17,18,19,20,21,22,23,24,25,26,27,28,29] and blood oxygen saturation (SpO_2_) [30,31,32,33,34,35,36,37,38,39,40,41,42,43,44,45,46,47,48,49,50,51,52,53,54,55], which are measured by a pulse oximeter. In some cases, the single-lead ECG signal is combined with the SpO_2_ signal [56,57,58,59,60,61].

Standard Heart-Rate Variability (HRV) mirrors the autonomic neural regulation of the heart and the circulatory system, and thus, changes are a result of different physiological factors modulating the normal heart rate (HR) [62]. HRV studies are carried out using the time elapsed between two successive R waves obtained by measuring the delay between two consecutive R-peaks of the ECG. The RR series is the sequence of consecutive delays. There is a strong physiological relationship between HRV and OSA [5]. During normal sleep, parasympathetic and sympathetic control produce cyclic oscillations in the RR intervals due to breathing phases (cardio-acceleration during inhalation and cardio-deceleration during exhalation) called respiratory sinus arrhythmia (RSA) [5]. In general, HRV frequency components are found between 0 and 0.5 Hz, and the RSA component stands out at the breathing frequency, which is approximately 0.25 Hz. However, during an apneic episode, increased sympathetic control in the cardiovascular system modifies this cyclic oscillation pattern, and we find a brady-tachycardia pattern called ‘cyclic variation of heart rate’ (CVHR) [5]. As a result of apnea repetition, the CVHR frequency components appear in a frequency band ranging from 0.01 to 0.04 Hz [20]. However, it is worth noting that HRV is influenced not only by apnea but also by many additional factors, such as sleep stages, other diseases, or medication, which may mask the CVHR pattern [19].

The information contained in the SpO_2_ signal is also valuable for apnea detection purposes, as it directly tracks the variations that typically take place in sleep apnea patients during apneic events. Indeed, the American Academy of Sleep Medicine Task Force [5] includes oxygen saturation as one of the conditions that characterizes obstructive sleep apnea–hypopnea events. Oximetry levels in healthy adults range between 96% and 99% and tend to remain constant, varying slightly with age and regardless of ethnicity, sex, or weight [32,55]. Repeated apneic events usually lead to recurrent hypoxias and hypercapnias. Therefore, a breathing disruption in sleep apnea usually results in an episode of hypoxemia that can be detected through a decrease in SpO_2_. This drop starts approximately 10 to 30 s after the apneic episode begins (slow desaturation) and normally starts to recover shortly after hypoventilation ends (fast resaturation) [5]. All this adds up to more irregular oximetry recordings in OSA patients with a typical saw-tooth morphology [49]. Moreover, an apneic episode lasts between 30 s and 2 min, including the arousal, and repeated apneic events lead to cyclical oxygen desaturations that take place with the same frequency. Thus, the signal power of the frequency band, ranging from 0.01–0.033 Hz, is usually higher in OSA patients [33] and is especially interesting for diagnostic purposes [40].

However, the SpO_2_ has an important drawback, as it has, in general, high specificity in sleep apnea, but the sensitivity is typically lower [56,63]. This is because breathing disruptions may not come accompanied by oxygen desaturations, especially if they are short [58,64,65]. Increased upper airway resistance may also lead to the absence of oxygen desaturations [66]. On the other hand, desaturations may also occur due to other pulmonary and cardiovascular diseases, such as chronic obstructive pulmonary disease (COPD) or alveolar hypoventilation [67,68]. Thus, patients who suffer from COPD and OSA simultaneously may experience more oxygen desaturations than those with only one of the two conditions [54]. Hence, features obtained from the SpO_2_ signal may not exactly mirror the apneic episodes, which could affect the performance of oximetry analysis [6]. Moreover, oximeters can also register poor quality SpO_2_ signals due to artifacts caused by motion or poor perfusion [58,69]. These can be considered the main handicap in exclusively using oximetry signals to detect apneas. Several authors have discussed this disadvantage in their works [34,42,58,64]. In Section 4.3, we provide a thorough analysis of several factors that can lead to non-desaturating apneas, which reinforce how important it is to avoid basing OSA diagnosis exclusively on the SpO_2_ signal.

### 1.2. Review of Relevant Literature

As OSA is considered to be a major health problem, several studies over the last 20 years have focused on addressing two main goals: automatic sleep apnea diagnosis (per-recording classification) and real-time detection of sleep apneic events (per-segment classification or quantification). In this section, we will focus on those studies in the literature that analyze extracting features from two of the most popular signals: the single-lead ECG and the oximetry signal.

As far as the single-lead ECG is concerned, a wide variety of feature extraction techniques have been followed to detect the pathological patterns underlying sleep apnea. On the one hand, linear analysis has been put forward to detect information about the periodicities taking place in the RR series [19,70,71]. On the other hand, nonlinear methods have been introduced to reveal the complex autonomic and respiratory control mechanisms that interact in the regulation of the cardiac function [18,19,27,72,73,74,75,76,77]. The reason is that the cardiovascular system and, by extension, the HRV are considered dynamic, nonlinear, and nonstationary [78,79]. Table 1 shows some relevant studies related to apnea detection using the single lead ECG.

Concerning the use of the SpO_2_ signal in apnea detection, several authors have also carried out works aiming to obtain further knowledge on oximetry dynamics associated with OSA, thus improving the diagnostic ability of overnight oxygen saturation monitoring. There are approaches that search for temporal OSA patterns [34,43,52,54], while others introduce spectral analysis to detect the peak commonly present in the 0.010 to 0.033 Hz frequency range during apnea [30,40,42,49,54]. More sophisticated techniques, such as nonlinear analysis, have also been proposed [34,41,47,80,81]. Recently, other techniques based on deep learning have also been presented [82,83]. The first studies that focused on sleep apnea detection by means of SpO_2_ used the conventional time-domain oximetry indices [36,84]. However, these methods are not suitable for real-time analysis, as they only identify OSA patients from whole overnight recordings. In the most recent works, conventional indices were included for comparison purposes with newer techniques [54]. Zamarrón et al. [30] were the first authors who introduced spectral analysis. They concluded, as did most authors who applied this technique, that, in general, spectral analysis and, in particular, the frequency content of the apnea frequency band (0.010 to 0.033 Hz) are especially suited for apnea detection [31,35]. Beyond these temporal and spectral features, there are also studies which propose time-domain and frequency-domain statistics. Álvarez et al. [85] reached, using the variance, the best performance within the time-domain statistics in the single feature evaluation. They concluded that the conventional features from spectral analysis perform, in general, better than other approaches. However, frequency-domain statistics achieve lower performance. The nonlinear features proposed in the literature for sleep apnea detection using the SpO_2_ signal include Poincaré parameters [50], approximate entropy (ApEn) [31,32,33,34,38,46,80], sample entropy (SamEn) [45], central tendency measure (CTM) [31,32,33,45,46,47,48], and Lempel–Ziv complexity (LZC) [31,32,33,34,38,45].

There are two groups of studies in which the single-lead ECG signal is combined with the SpO_2_ signal [56,57,58,59,60,61]. In the first, research focuses on AHI estimation and, thus, on OSA diagnosis (per-recording classification) [56,86]. Raymond et al. [56] estimated the number of respiratory event-related arousals by counting the number of autonomic arousals (based on changes in the heart interbeat interval) that coincided with a rise in oximetry. They collected an hourly index that was defined as the cardiac-oximetry disturbance index (CODI) and assessed the correlation between the CODI and the AHI. LaFleur et al. [86] introduced an automated algorithm for detecting the presence of Long QT Syndrome (LQTS), which is associated with OSA. They also proposed a correlation classifier to correlate the ECG signal and the SpO_2_ data, resulting in an estimated AHI. In the second group, the studies carry out a real-time analysis aiming to detect apneic episodes on a minute-by-minute basis (per-segment classification) [57,58]. Heneghan et al. [57] put forward an algorithm that provided an estimation of apnea occurrences epoch-by-epoch and estimated an overall per-recording AHI. De Chazal et al. [58] proposed an algorithm to identify epochs as normal or sleep-disordered breathing and to classify the pathological ones into one of six classes: obstructive, mixed, and central apnea, as well as obstructive, mixed, and central hypopnea. They derived an AHI estimation from this epoch-based classification approach using frequency and time domain features from the ECG signal, as well as time-domain oximetry features.

### 1.3. Objectives of This Study

In Section 1.1, we highlighted the limitations of using single-lead ECG and oximetry signals, individually, to detect sleep apnea. There are several articles devoted to the study of combining both signals for diagnostic purposes. However, to the best of our knowledge, until now there has been no work reporting improved performance when these signals are used alone vs. combined in patients who do not show marked oxygen desaturations during apneic events, hereafter referred to as non-desaturating patients. In the literature, authors only refer to non-desaturating patients within the limitations of the performed studies. In our analysis, we include a dataset that clearly distinguishes, among control, desaturating, and non-desaturating patients, the HuGCDN2014-OXI database, thus allowing a thorough quantitative analysis of our system under different circumstances. Moreover, we assess the performance of our system using the popular Apnea-ECG Physionet database. As for meaningful comparison with prior work, results obtained from the same database must be compared. Including both databases makes the results more generalizable, and thus, conclusions would not be limited by database variability.

Thus far, there is no agreement about which features best describe the underlying process when apnea occurs. That is why there is still huge pressure for new studies to better understand the physiological mechanisms associated with sleep apnea that are mirrored in HRV and SpO_2_. If we only took into account the saturation information to detect respiratory events, we would find that, in some events, there is no desaturation, both in apneas and hypopneas. That is why we hypothesize that we can improve the detection of respiratory events using the information contained in the HRV and, thus, get closer to the AHI values.

Therefore, and according to our previous research [22,61,87,88], we propose a feature combination that includes measures analyzed independently and that also had good results. We specifically measure the discriminant capacity of feature combinations from HRV and oximetry to assess its performance compared with that using oximetry-based features alone, especially in non-desaturating patients.

**Figure 1:**
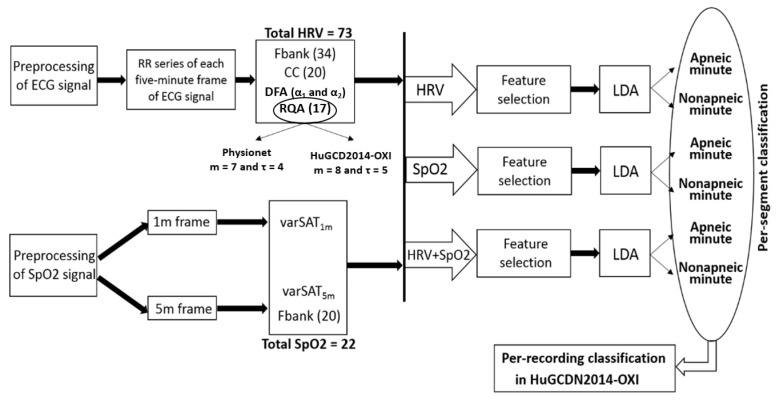
Graphical representation of the procedure followed in the present work.

Figure 1 shows a graphical representation of the system followed in the present work.

## 2. Materials and Methods

### 2.1. Databases

We used two databases to carry out the experiments: the Apnea-ECG Physionet database, hereinafter referred to as Physionet database, and the HuGCDN2014-OXI database.

#### 2.1.1. Physionet Database

The Physionet database, available free of charge from the Physionet website [89], was provided by Prof. Dr. Thomas Penzel for Computers in Cardiology Challenge 2000. Since then, it has been widely used for sleep apnea studies. The eight recordings include single-lead ECG (digitized at 100 Hz) and SpO_2_ signals (digitized at 8 Hz), as well as their corresponding scores, on a one-minute basis. Therefore, our real-time system is designed to give a minute-by-minute result. A physician, based on simultaneously recorded respiratory signals, performed the labeling process. The recordings lasted approximately 8 h. Of the eight recordings, five are sleep apnea patients with diverse AHI values, and three are control subjects. Demographic characteristics are summarized in Table 2. More details about the data are available in [89].

In each of the databases, we must define the learning set (L-set) used in the training process and the test set (T-set), for the assessment of the system. Concerning the Physionet database, we have used a crossvalidation strategy in the assessment process, as only 8 patients are available, which has allowed us to reach statistically valid classification results [38]. In each iteration, the system is trained with the L-set, made up of three OSA patients and two control subjects, and assessed with the remaining patients (T-set).

#### 2.1.2. HuGCDN2014-OXI Database

**Figure 2:**
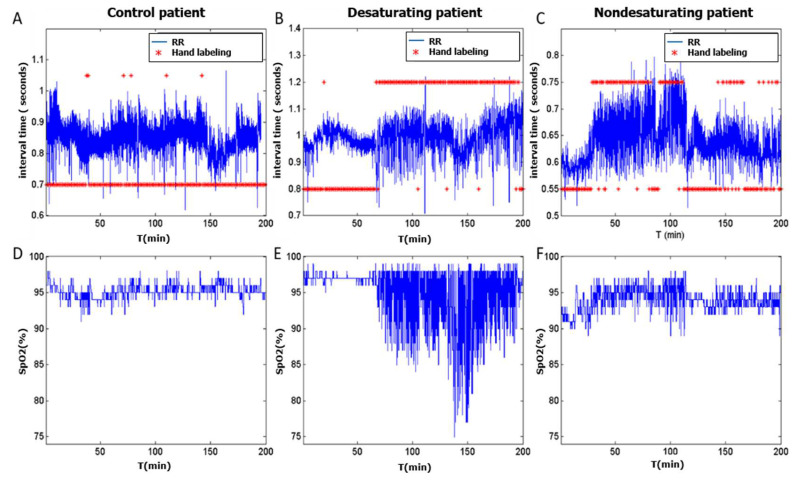
RR and SpO_2_ signals of a control subject (**A**,**D**), a desaturating patient (**B**,**E**), and a non-desaturating patient (**C**,**F**). Within the hand labeling, the red asterisks at the top represent apnea minutes, and the red asterisks at the bottom represent nonapnea minutes. The behavior of the oximetry signal in desaturating and non-desaturating patients differs significantly.

The HuGCDN2014-OXI database was provided by the Dr. Negrín University Hospital (Las Palmas de Gran Canaria, Spain) and contains recordings of 83 subjects, each containing the ECG signal, digitized at 200 Hz, and the SpO_2_ signal, digitized at 50 Hz. The sleep studies and the labeling process were carried out following the American Academia of Sleep Medicine (AASM) guidelines [5]. As in the Physionet database, a physician labeled every minute based on simultaneous polysomnography, marking it as apnea or nonapnea. There are three groups: (1) CONTROL: 38 healthy subjects with AHIs lower than 5 (range, 0–5); (2) DESATURATING PATIENTS: 34 OSA patients with AHIs higher than 25 (range, 30–106.3) who show desaturations during apneic episodes; 3) NON-DESATURATING PATIENTS: 11 OSA patients with AHIs higher than 25 (range, 26.2–87.5) who do not always show desaturations during apneic episodes. Patients are defined as non-desaturating when their ODI is less than half their AHI. In our case, 75% of the non-desaturating patients present an ODI less than 7.35 and an apnea total of less than 98, i.e., for 75% of the non-desaturating patients, the percentage of desaturating apneic events is below 7.5%. Demographic characteristics are summarized in Table 3. Figure 2 shows the RR and SpO_2_ signals of all types of subjects: control, desaturating, and non-desaturating. Hand labeled apnea events are depicted as red asterisks. As we can see, the behavior of the oximetry signal in desaturating and non-desaturating patients differs significantly.

The performance of the system for the HuGCDN2014-OXI database has been assessed using three testing sets of patients. Before designing these sets, we have defined the L-set, which is composed of half of the control and half of the desaturating patients. The test sets are: (1) T1-set—half of the desaturating patients, none of whom is included in the L-set; (2) T2-set—all non-desaturating patients; (3) T3-set—composed of three subsets: half of the control patients excluded in the L-set, patients in T1-set, and patients in T2-set.

### 2.2. Signals Preprocessing

The ECG signal is divided into five-minute segments that are shifted, in time, in increments of 1 min [19,21,23,26,28,90,91]. This frame length suits our analysis because CVHR oscillations vary between 20 and 60 s, and CVHR recurrence is only recognizable if there are several oscillations in the frame [90]. There is also a good balance between stationarity and good spectral resolution. The quantification obtained for each frame is assigned to the central minute, thus achieving the scoring on a one-minute basis.

For each five-minute segment, the RR series is constructed as the sequence of delays between consecutive R-peaks of the ECG. The R-peak detection is inspired by the Pan–Tompkins algorithm [92]. After the creation of the RR series and before obtaining the corresponding features, it is necessary to apply a procedure that automatically removes artifacts and ectopic values. We used the method proposed by Wessel et al. for this purpose [93]. The main advantage of this algorithm is that the adaptive filtering procedure spontaneously adapts the filter coefficients to sudden changes in the RR series.

As far as the SpO_2_ signal is concerned, artifacts detected are mainly related to signal interruptions due to poor contact between the finger and the sensor or due to a patient’s movements, which appear as zero levels in the oxygen saturation. An automated algorithm eliminates the zero values to remove them from the signal. Moreover, in the same process, oxygen saturations below 50% are also considered artifacts, and they are removed from analysis [38,42,60]. Finally, the mean is subtracted from the SpO_2_ signal [49,50]. The average number of segments over each of the signals for each subject is 407 in HuGCDN2014-OXI (from 302 to 474) and 495 in Physionet (from 455 to 529).

### 2.3. Feature Extraction

#### 2.3.1. Oximetry Features

For the oximetry analysis, we extract both time-domain and frequency-domain features, which provide complementary information about sleep apnea episodes.

(a) Time-domain features:

Oximetry is, under normal circumstances, a very stable signal. However, sleep apnea events are commonly related to hypoxemias and, thus, to drops in the SpO_2_ values. These changes are mirrored in the signal’s variance, which can be taken as a valuable measure to uncover breathing disruptions. To decide the frame length to consider in the variance analysis, it is important to take into account that a breathing pause is at least 10 s long and can last over 50 s [5]. Therefore, 1 min segments are suitable for our study, as they detect short and long apneic events. However, OSA patients may also have consecutive apneic episodes. To consider this possibility, we include the 5 min variance in the analysis. Thus, both variances are defined as varSAT1m and varSAT5m [61]. In the latter case, as in the ECG signal, we consider 5 min segments, shifted in time in increments of one minute, and the variance is assigned to the central minute.

(b) Frequency domain features:

As the signal power in the frequency band, ranging from 0.01 to 0.033 Hz, is usually higher in OSA patients [33], spectral features are especially interesting for diagnostic purposes. In our study, we obtain the power spectrum of the SpO_2_ signal by means of the periodogram based on the Discrete Fourier Transform (DFT), according to Equation (1),

(1) $$S(k)=\frac{1}{N}{\left|X_{N}(k)\right|}^{2}$$

where *X_N_*(*k*) is the DFT of the analyzed signal, and *N* represents the number of samples. The samples of periodogram, *S*(*k*), are obtained for each 5 min oximetry segment, with 1 min of displacement between adjacent frames. To evaluate the information contributed by each frequency band, we introduce a filter bank (Fbank-FbSAT) implemented directly in the transformed domain [61]. We call *M* the number of filters in the filter bank, and we have as many parameters as filters in the bank. The outcomes are an estimation of the normalized power in each frequency band. As in the 5 min variance, we assign these parameters to the central minute of the 5 min segment. According to previous research [61], *M* = 20 is considered a suitable value for the number of filters for the oximetry signal. In short, 22 features will represent every minute of the SpO_2_ signal.

#### 2.3.2. HRV Features

For each frame, we carry out frequency domain (Fbank-FbHRV) [18,22], cepstral domain (cepstrum coefficients-CC) [18,22,94], and nonlinear analysis. The latter includes DFA [18,22] and RQA [87]. The feature vector representing each frame contains the combination of all the previous measures.

(a) Frequency domain features:

As in the oximetry signal, the frequency band of interest to detect the CVHR components, due to apnea repetition, ranges from 0.01 to 0.04 Hz [20]. The procedure that followed to extract spectral information is the same as in the SpO_2_ signal (explained in Section 2.3.1 (b)). The only difference is that, according to the results in previous research [18,22], the number of filters in the filter bank is set to *M* = 34, as this value gives the frequency content analysis enough resolution, especially the CVHR and RSA components.

(b) Cepstral domain features:

Cepstral Analysis also detects periodic structures in signals, which appear as peaks in the cepstral representation. The cepstrum coefficients (*c*(*τ*)) are found by taking the inverse Fourier transform (*F*^−1^) of the logarithm of the magnitude of the RR spectrum (*F*(*x*(*n*))), according to Equation (2). In the experiments, we introduce the real cepstrum, which uses the spectrum’s magnitude only, thus obtaining information about the spectrum envelope, as well as the harmonic components.

(2) $$c(\tau )=real\left(F^{-1}\left(\log(\left|F(x(n))\right|)\right)\right)$$

From the cepstral coefficients, we take the first 20 elements that we found in previous work to contain relevant information of the underlying system [18,22].

(c) Detrended Fluctuation Analysis (DFA):

It is well known that RR series normally fluctuate in a complex, apparently erratic manner [95]. A question we have faced is whether this heterogeneous structure arises from the intrinsic dynamics of the heart rate or if fluctuations arise from a complex, nonlinear, and dynamic behavior. This question has attracted our attention and is the reason we applied it. In previous works [18,22], we have found that the heartbeat time series is often highly non-stationary, and we have seen that the physiological process associated with OSA can be characterized. In this context, DFA, which was first introduced by Peng et al. in 1995 [96], is a good option to characterize the physiological process associated with OSA and to detect apneic events.

RR series may possess long-memory structure, so DFA is well-suited as a scaling analysis method. There are two main properties that make DFA especially valuable for detecting apneic episodes [18,19,22]: (1) it can be applied to stationary and nonstationary signals, and (2) it avoids spurious detection of long-range correlations in RR interval series that are artifacts of nonstationarity [19].

The DFA parameters are approximated by power-law (3) [18,19,22,95,96,97,98]:

(3) $$F(t)∼t^{\alpha }$$

where *α* is called the scaling exponent. The scaling exponent is also the slope of the line relating log *F*(*t*) to log *t*. For uncorrelated data, *α* = 0.5.

We have paid attention to the analysis of short-range and long-range correlations in the RR series, which help discriminate apneic and nonapneic minutes. We introduce two different scaling exponents. These are *α*_1_ (short-range correlations, for time scales *t* between 10 and 40 beats) and *α*_2_ (long-range correlations, for time scales *t* between 70 and 194). The limits for the time scales are based on previous work [18,19,22].

(d) Recurrence Quantification Analysis (RQA):

A recurrence plot (RP), first proposed by Eckmann et al. [99], is a two-dimensional plot that represents a binary symmetric square recurrence matrix. An RP is useful to visualize the recurrence characteristics of systems. This matrix shows the times when two states can be considered neighbors in the phase space, according to a certain threshold [100]. It is considered especially useful to uncover hidden periodicities and characteristics that, otherwise, would remain unnoticed [101].

The first step prior to constructing the RP of a time series, *u*(*t*), is embedding. The most widely used strategy to carry out this process is the Takens time delay method [102]. This procedure aims to reconstruct a multivariate phase space that represents the original system from a single measured variable of that system [100]. It, therefore, generates time-delayed copies of the variable under study, so the original time series is considered a dimension of the underlying system, and each of its delayed copies becomes a new dimension of the system. A phase state, ${\vec{x}}_{i}$, is defined as follows Equation (4):

(4) $${\vec{x}}_{i}=\left[u(i),u(i+\tau ),\ldots ,u(i+(m-1)\cdot \tau )\right]$$

where *τ* is the time delay and *m* is the embedding dimension. The delay is chosen to achieve variable independence, while avoiding state vectors that are autocorrelated [103]. The dimension represents the number of independent variables influencing the system under study. The delay is commonly chosen by calculating the first local minimum or the first zero crossing of the autocorrelation or mutual information [100]. For the dimension, the false nearest neighbors (FNN) method is the most widely used. A neighbor is considered a false neighbor when it is defined as a neighbor only because the dimension of the state space is too small. The dimension is increased in integer steps until the number of false nearest neighbors drops to zero [104]. In our experiments, and according to previous work [87], we concluded that working with dimensions of approximately 7–8 and delays of approximately 4–5 yield the best results in two different datasets, supporting the idea that reached results are potentially generalizable. In this work, the values for dimension and delay are *m* = 7 and *τ* = 4 for the Physionet database and *m* = 8 and *τ* = 5 for the HuGCDN2014-OXI database, which were considered optimal for the same databases in previous work [87].

After embedding, the RP is constructed according to the following equation:

(5) $$R_{i,j}=\Theta (\varepsilon_{i}-\left\|{\vec{x}}_{i}-{\vec{x}}_{j}\right\|),\;\;\;\;\;\;\;i,\;j=1\;,2\;,\ldots ,\;N$$

where *N* is the number of reconstructed points ${\vec{x}}_{i}$, *ε* is the threshold distance, Θ is the Heaviside function (Θ(*x*) = 0 if *x* < 0 and Θ(*x*) = 1, otherwise), and ||⋅|| is the norm [101]. First, we construct a distance matrix (DM), and afterwards, we apply a cutoff distance to find the recurrence matrix (RM). This process results in an *N* × *N* symmetric matrix, containing *R_i,j_* = 1, if ${\vec{x}}_{i}$ and ${\vec{x}}_{j}$ are neighbors, according to the ε-threshold, and *R_i_*_,*j*_ = 0, if not. The RP is the graphical representation of the RM. For stochastic or chaotic signals, RPs are formed by isolated points with no, or very short, diagonal structures, whereas periodic and deterministic signals show longer diagonals with less single recurrence points [105].

Apart from the embedding parameters, delay, and dimension, the other crucial parameter is the distance threshold in the RP is *ε*. There are two approaches to define it. We can either choose a fixed value so that *ε_i_* = *ε*, called the Fixed Distance Method, or we define this parameter so that each point of the trajectory is surrounded by a constant number of neighboring states, i.e., *ε_i_* changes for each state, called Fixed Amount of Nearest Neighbors (FAN) Method. The latter case results in a constant density of recurrence points in each column [106]. Although, in general, the Fixed Distance Method is used more often than the FAN Method, we introduce the FAN Method with 5% of neighbors, according to previous results [87], this approach greatly improves the system performance using the Fixed Distance Method.

After constructing the RP, we obtain the RQA measures. RQA is the quantitative analysis of RP structures that allows us to obtain information about the system dynamics. There are features based on the recurrence density, as well as on the diagonal, vertical, and horizontal line structures that appear in the RP [101]. Moreover, another group of features can be derived from RPs, which are related to recurrence times [107]. Finally, we also include new measures originating in the complex network theory, such as clustering coefficient or transitivity [108], which, when applied to recurrence matrices, are more powerful and reliable for detecting periodic dynamics [109,110]. In our analysis, we found 17 features for each RR 5 min frame. Table 4 shows the RQA features included in the feature set. In our experiments, we used the Cross Recurrence Plot Toolbox (CRP Toolbox) [111].

In summary, 73 features will represent every 5 min RR frame (spectral analysis: 34, DFA: 2, cepstrum: 20, and RQA: 17).

### 2.4. Feature Selection

After preprocessing and obtaining the features of both signals, HRV and SpO_2_, we carry out a feature selection process. This procedure helps us find a reduced feature set, made up of those variables that differentiate the two classes best, i.e., apneic and nonapneic minutes. Furthermore, this technique has other advantages: (1) it prevents overfitting by ruling out redundant and irrelevant features, (2) it reduces computational load, and (3) the selection facilitates the physiological interpretation of the results. In our analysis, we use a repeated random sub-sampling validation that leads to dimensionality reduction and increased accuracy [22,61,87,94,112]. This method evaluates the performance of a feature group, taking into account their interactions, unlike other feature selection techniques proposed in the literature, which only assess individual features [17,29,113]. We carry out the feature selection algorithm for the three different feature combinations: all HRV variables (73), all SpO_2_ variables (22), and the whole set of HRV and SpO_2_ variables (95).

**Figure 3:**
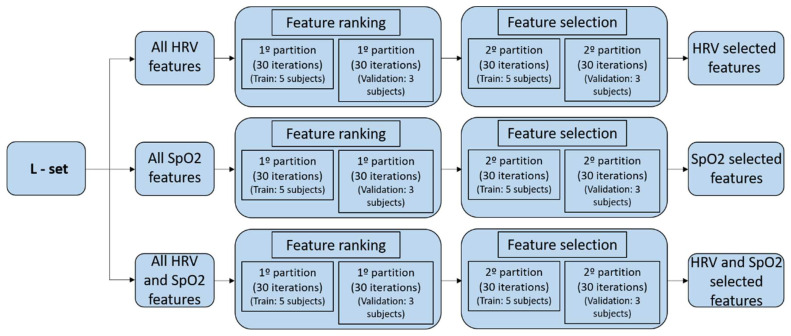
Graphical representation of the feature selection process in the Physionet database. In the procedure, we only use the learning set (L-set) defined for Physionet.

**Figure 4:**
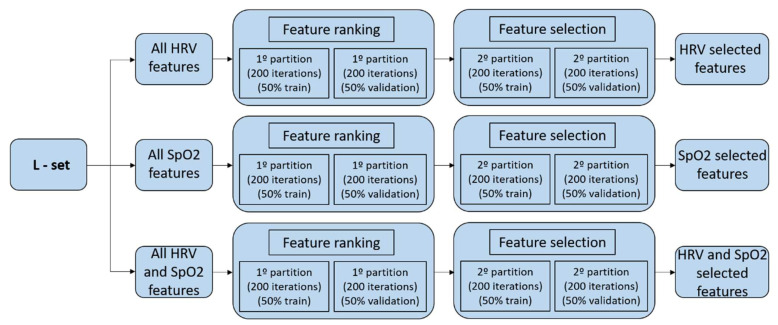
Graphical representation of the feature selection process in the HuGCDN2014-OXI database. In the procedure, we only use the learning set (L-set) defined for HuGCDN2014-OXI.

In the procedure, we only use the learning set (L-set), as defined for each of the databases in Section 2.1. The graphical representation of the feature selection process is shown in Figure 3 and Figure 4 for both databases. In the HuGCDN2014-OXI database, the L-set is divided into two equally sized groups that form a training set and a validation set, each containing the feature vectors of the randomly selected patients in each iteration. Thus, we avoid feature vectors from one patient being in both the training set and the validation set simultaneously. For this dataset, the number of iterations is set to 200, as this value provides stable results. In the Physionet database, as the training set is always made up of three OSA patients and two control subjects, and the validation set is the rest of the 8 subjects, the total number of possible combinations is 30. That is why there are 30 iterations in this database.

The whole process is divided into two steps. First, we create the feature ranking according to the number of times a certain feature is selected by the sequential forward feature selection method (based on the classifier performance). In each iteration, the optimal feature set corresponds to the maximum accuracy in the validation set. Second, repeating a random sub-sampling validation process again, we obtain the error rate for an increasing number of features. They are entered in the same order as they appear in the ranking created in step one. This procedure assesses the averaged misclassification error, obtained for the validation data, while increasing the number of features. The final selected features will be those with the minimum averaged misclassification error. In all cases, the number of features selected is smaller than the original number of features.

### 2.5. Classifiers

The detection of apneic episodes is carried out on a minute by minute basis, thus allowing real-time analysis. The proposed classifier is LDA, widely used for apnea detection and with good results [24,31,33,61]. Moreover, LDA also balances performance, complexity and interpretation, unlike other pattern recognition techniques that require setting different model parameters by the user, leading to a more complex training process [31]. We balanced the classes using an under-sampling strategy [114].

## 3. Results

The main objective of the data analysis presented below is to evaluate the benefits found, both for the minute-by-minute classification of apneic events and for the overall classification of patients. In both cases, we focus, especially, on what happens when the subjects affected by OSA do not present clear desaturation patterns (non-desaturating patients). Likewise, we analyze the selected characteristics when using HRV variables, SpO_2_ variables, and when both types are combined. This procedure evaluates the relative importance of each one in the detection of apneas and, indirectly, in the characterization of the physiological system associated with this phenomenon. The measures considered for the system evaluation include classification rate, sensitivity, specificity, and AUC (Area Under Curve), where curve refers to ROC (Receiver Operating Characteristic).

Within these parameters, sensitivity is considered especially relevant in the medical setting [32]. Sensitivity reflects the ability to correctly detect apneic minutes, within quantification, and OSA patients in per-recording classification. However, specificity shows the ability of the system to distinguish normal minutes and healthy patients, respectively. There is usually a compromise between both values, namely sensitivity and specificity. However, for the diagnosis of OSA or the detection of apneic episodes in general, we are more interested in a high sensitivity that reduces the risk of false negatives, as the impact of incorrect classification on a patient diagnosed with OSA will be greater than if the classification is incorrect in healthy patients [49,60]. This is especially important in this type of pathology, given the serious long-term health consequences. On the other hand, the AUC values will allow us to evaluate the system’s performance, regardless of the working point (threshold) selected on the ROC curve.

### 3.1. Per-Segment Classification and Characteristic Selection

As pointed out in the previous sections, the considered HRV characteristics include those gathered through spectral analysis (FbHRV), cepstral analysis (CC), and nonlinear analysis (two DFA variables and 17 RQA variables). On the other hand, oximetric characteristics include variables corresponding to spectral analysis (FbSAT) and two variables related to temporal signal analysis (varSAT_1m_ and varSAT_5m_). The features, selected according to the procedure shown in Section 2.4, are the input of a LDA classifier. In both databases, the results correspond to the point on the ROC curve with the shortest distance to the upper left vertex.

Table 5 contains the test results obtained with the Physionet database using crossvalidation. Table 6, Table 7 and Table 8, corresponding to the HuGCDN2014-OXI database, distinguish between three cases: test results of desaturating patients (T1 test set) are shown in Table 6, results of non-desaturating patients (T2 test set) are in Table 7, and results of the whole set of control subjects, desaturating, and non-desaturating patients (T3 test set) are in Table 8. Each of the Table 5, Table 6, Table 7 and Table 8 shows results attained by introducing only HRV characteristics, only oximetric characteristics, and by combining them.

The first database, Physionet, does not distinguish between desaturating and non-desaturating patients. However, as it is one of the most widely used databases for sleep apnea studies, the results obtained will allow us to compare the performance of the proposed system with other existing systems in the literature. In general, we can conclude from Table 5 that the system performs very well with this database. As far as the AUC values are concerned, in all cases, they are higher than 0.98. Namely, results indicate that only six oximetric characteristics provide slightly better performance than the seven HRV characteristics. When we combine the two types of characteristics, there is another increase in the AUC, but it is at the cost of increasing the number of variables to 12. The classification rate and sensitivity behave similarly to the AUC. However, in this case, the increase when applying the oximetric characteristics, both in the classification rate and in the sensitivity, is approximately 3%, compared to those reached by the HRV characteristics. Moreover, the improvement obtained by combining both types of variables does not, in any case, exceed 1% compared to the values shown for SpO_2_ characteristics. The previous analysis indicates that combining electrocardiographic and oximetric characteristics yields the best results with 12 variables. However, the improvement is so small that, in this case, the introduction of the two signals would not be justified. Therefore, for this database, we would propose the use of six oximetric features.

However, these results cannot be considered conclusive given the limited number of patients under study.

Nonetheless, studying Table 6, Table 7 and Table 8, belonging to the HuGCDN2014-OXI database, we can explore the real purpose of this analysis, as it distinguishes between desaturating and non-desaturating patients. The first two tables (Table 6 and Table 7) contain the results for each type of patient (desaturating and non-desaturating). Finally, Table 8 shows the results representing a real system, in which the tested subjects can be healthy or diagnosed with OSA (desaturating and non-desaturating).

Table 6, corresponding to desaturating patients, shows that the classification rates are lower when only HRV features are used. When we consider oximetric variables or combine both types, the results are very similar, reaching values that are approximately 10% higher than those reached only with HRV. The sensitivity results are high (>82%). As with the classification rates, the oximetric variables, or the combination of oximetric and HRV variables, provide the best results for sensitivity (higher than 97%). Finally, AUC shows a similar behavior. There is an approximate 10% increase when using SpO_2_ features instead of HRV variables. When both types are combined, the value reached is practically the same as the values gained with only SpO_2_. Therefore, in this case, oximetric features are the most appropriate because, similar to the HRV + SpO_2_ combination, it uses 24 characteristics instead of 15 (SpO_2_).

Table 7 shows the results obtained for non-desaturating patients. According to classification rates and AUCs, we can conclude that the values are lower than those reached in desaturating patients (Table 6). This indicates that the detection of apneic events is more difficult in non-desaturating patients than in desaturating ones. Moreover, there is a progressive increase in the results, between 2 and 5%, when using HRV features, SpO_2_ features, and a combination of both types. This behavior also differs from that observed in Table 6. However, the most noteworthy aspect is related to sensitivity. HRV features provide the best values (72%). These results contrast with those obtained for desaturating patients, where HRV features yielded the lowest sensitivities and classification rates. However, when we include SpO_2_ variables in non-desaturating patients, sensitivity drops considerably, as expected. Nevertheless, the most relevant result is related to the increase in sensitivity, from 51.24% to 59.79%, when using HRV + SpO_2_ features instead of SpO_2_ features alone.

As stated above, Table 8′s results represent a real system tested with healthy subjects and OSA diagnosed patients (desaturating and non-desaturating). For the main parameters (classification rate, sensitivity, and AUC), using only the HRV features yields the worst results, which improve using only SpO_2_ variables, but we obtain the best results by combining both feature types.

**Figure 5:**
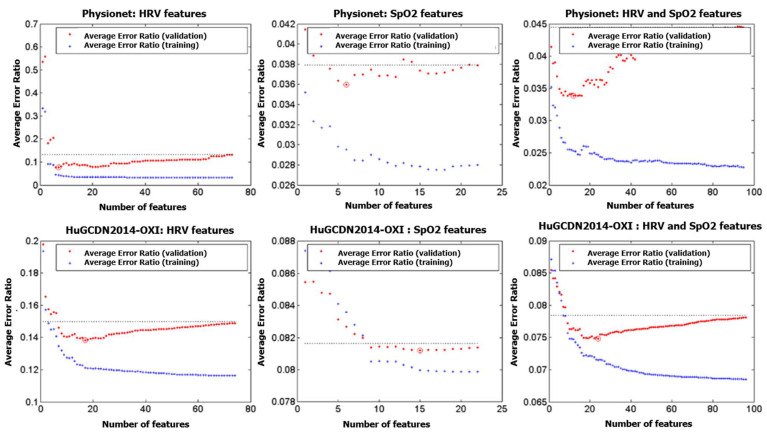
Evolution of the averaged error rate to the number of selected features for both training and validation. The horizontal dotted line represents the misclassification error obtained without feature selection, and a circle indicates the point with the minimum validation error rate.

For the feature selection process, Figure 5 shows the evolution of the averaged error rate to the number of selected features for both training and validation (see Section 2.4). The horizontal dotted line represents the misclassification error obtained without feature selection, and a circle indicates the point with the minimum validation error rate. What is particularly interesting is that there are cases with a clear minimum, as in Physionet with SpO_2_ features, and other cases in which, for a given range of features, the error rates are very similar, as in Physionet when HRV and SpO_2_ features are combined. In the latter cases, we could, therefore, reduce the number of features indicated in the third column of Table 5, Table 6, Table 7 and Table 8, since with a smaller number of variables, results would be very similar. The column headers of Table 9 (Physionet) and Table 10 (HuGCDN2014-OXI) show the number of features for each case. We only see one value if there is a clear minimum. However, if we can reduce the number of variables, we indicate it with an arrow and the reduced number. In the latter cases, thicker lines delimit these variables in their respective columns.

Table 9 and Table 10 also show the selected features after the feature selection process for both databases. The order in which they appear is in agreement with the feature ranking created according to the number of times a certain feature is selected by the sequential forward feature selection method. The three columns contain the chosen variables when HRV, SpO_2_, or HRV + SpO_2_ features are used. Regarding HRV, the algorithm proposes the combination of variables containing spectral, cepstral, and non-linear information (both DFA and RQA). Although the number of selected features in both databases is different, some are always in the final set, namely the first and fourth cepstrum coefficients, the α_1_ variable (DFA), representative of short-term correlations, and T2 and Trans (RQA).

However, for spectral information, while both low frequency information, represented by FbHRV 1 and 2, and higher frequencies (FbHRV 11 and 15) are considered relevant in the HuGCDN2014-OXI database, only one variable referring to higher frequencies (FbHRV 21) is included in Physionet. That is why we understand that all the information contained in these characteristics, especially the cepstral and non-linear information (DFA and RQA), plays an important role in the classification process and, therefore, in the characterization of the physiological system underlying sleep apnea.

Using the SpO_2_ features yields similar results in both databases. However, as we saw previously, the number of characteristics selected in the HuGCDN2014-OXI database is greater than in Physionet. The selected variables always include signal variances (varSAT1m and varSAT5m) and spectral information: in particular, the very low frequencies (FbSAT 1 in Physionet, and FbSAT 1 and 2 in HuGCDN2014-OXI), two intermediate bands (1st band: FbSAT 4 in Physionet, and FbSAT 5 in HuGCDN2014-OXI; 2nd band: FbSAT 12 in Physionet, and FbSAT 9 and 10 in HuGCDN2014-OXI), and a band of higher frequencies represented by FbSAT 17 in Physionet, as well as FbSAT 17 and 20 in HuGCDN2014-OXI. Thus, information related to low frequency oscillations, linked to consecutive apneic episodes, as well as some high frequency components related to short sleep breathing pauses, seem to be relevant.

Finally, we analyze the feature set obtained when we merge HRV and SpO_2_ variables. The selection of variables that refer to variance (varSAT1m and varSAT5m) in the first positions is especially noteworthy. Moreover, this selection includes features related to the spectral content of the oximetric signal, located in the bands referred to previously. However, RQA measures take on special relevance for variables related to HRV. Specifically, in Physionet, these include T2, Clust, TT, RT, and RTmax, and in HuGCDN2014-OXI, they include Clust, Trans, DET, T2, RT, LAM, TT, Lmax, and ENTW. Except for RTmax, all those chosen in Physionet are also chosen in HuGCDN2014-OXI. However, cepstrum coefficients are discarded in both databases. The feature selection algorithm also rules out the characteristics linked to the spectral content in Physionet, and only three (FbHRV 1, 23, and 30) occur in HuGCDN2014-OXI, and not in the first positions. Finally, the variable α_1_ (DFA) is discarded in Physionet, but it is included in HuGCDN2014-OXI and in a privileged position within the ranking.

We show the values and differences observed in the selected features in apneic and non-apneic minutes in Table 11 (Physionet) and Table 12 (HuGCDN201-OXI), where the medians and the interquartile ranges are defined. We applied the Wilcoxon test to compare statistical significance in the two groups under study. The test was performed using a level of significance *p* = 0.05, and values of *p* < 0.05 were considered significant. The corresponding *p*-values are shown in Table 11 and Table 12.

### 3.2. Per-Recording Classification

The aim of this last section is to evaluate the proposed system in OSA diagnosis, with special emphasis on those patients who do not exhibit clear desaturating behavior during apneic events. This is especially important given that the ultimate goal of any OSA automatic diagnostic system is to contribute to the search for an alternative to polysomnography. In this section, we will only refer to the HuGCDN2014-OXI database, as we only have eight patients in Physionet and, furthermore, it does not distinguish between desaturators and non-desaturators.

We carry out a per-recording classification according to the AHI estimation (automatic AHI). As we highlighted in our introduction, we estimate the AHI for a subject by calculating the average number of apneic minutes per hour, i.e., adding the total number of apneic minutes, dividing this value by the total number of minutes in the corresponding register, and multiplying the result by 60 [25].

**Figure 6:**
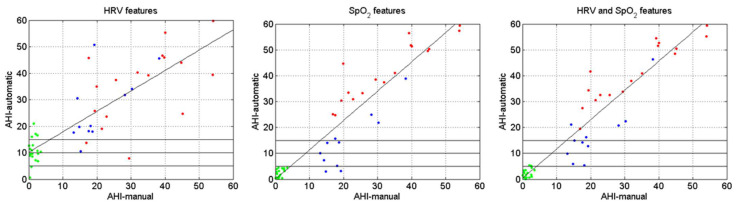
Automatic versus manual AHI (obtained manually by qualified staff) depending on the feature type (HRV, SpO_2_, HRV, and SpO_2_) in test patients (T3). The different colors represent the control patients (green), the desaturators (red), and the non-desaturators (blue). Horizontal lines represent AHI values commonly used in the OSA diagnosis: 5, 10 or 15.

Figure 6 shows automatic versus manual AHI (obtained manually by qualified staff) depending on the characteristics (HRV, SpO_2_, HRV, and SpO_2_). The different colors represent the control subjects (green), the desaturators (red), and the non-desaturators (blue). There is no defined criterion for the AHI value to discriminate between healthy and pathological subjects [44], so there are three horizontal lines in each graph representing AHI values commonly used in the OSA diagnosis—5, 10, or 15 [41,57]—thus calculating the results according to the AHI value. We perform the per-recording classification analysis graphically, as it is a good way to assess how our system’s regression line fits the function y = x.

Figure 6 shows that, using only HRV features, the limit for the AHI value should be set at 15. In that case, 4 healthy subjects (out of 19) and 3 pathological subjects (out of 28, 17 desaturators and 11 non-desaturators) would be incorrectly classified. Of the three pathological subjects, two are desaturators, and one is a non-desaturator. However, using only oximetric variables leads to a high specificity in the classification of control patients and a high sensitivity in the detection of desaturating patients. This means that, regardless of the AHI value (5, 10, or 15), all these subjects (control and desaturating patients) are well-classified. Nevertheless, of the 11 non-desaturating patients, and depending on the AHI limit, 2 for AHI = 5, 4 for AHI = 10, and 7 for AHI = 15 would be incorrectly classified. Finally, when we merge HRV and SpO_2_ features, sensitivity improves when classifying non-desaturating patients, and specificity worsens slightly. In this case, by setting the AHI limit at 5, almost all subjects would be well classified, although 3 subjects (2 control and 1 non-desaturator) would be at the limit. Accuracies, sensitivities, and specificities are shown in Table 13.

The regression study presented in the previous paragraphs is good to assess the agreement and relationship between manual (AHI_m_) and automatic (AHI_a_) AHI estimations. We now go a step further by performing a statistical analysis of the errors of the automatic method compared to the manual method. For this purpose, the quantification of the agreement between the two quantitative AHI measurements plays an important role by studying the mean difference and constructing limits of agreement. We are interested in evaluating the bias, which is calculated from the mean error, and estimating an agreement interval, within which fall 95% of the errors. Bland and Altman (B&A) introduced a plot to describe the agreement between two quantitative measurements [115,116]. The resulting graph is a scatter plot, in which the vertical axis shows the difference between the two-paired measurements (AHI_a_ − AHI_m_), and the horizontal axis represents the average of these measures, estimated as (AHI_a_ + AHI_m_)/2.

We can summarize the agreement by calculating the bias, estimated by the mean error ($\bar{d}$) and the standard deviation (s_d_). Assuming normality, we can expect 95% of the errors to lie in the confidence interval (CI) defined by $\bar{d}\pm s_{d}$. A bias is not statistically significant if the line of equality (zero error) lies inside the CI, thus indicating that there is not a significant systematic error. For example, the automatic method does not show a significant constant under/overestimation compared to the automatic one. We may wonder about whether the agreement interval is sufficiently narrow. It depends on analytical and clinical goals. For our purposes, correct classification must be as high as possible. It is also important to understand the significance of confidences around the mean error and agreement limits, as these CI describe possible errors in the estimates due to deficiencies in the sampling. In general, the greater the number of samples used to evaluate the quality, the narrower the CI.

**Figure 7:**
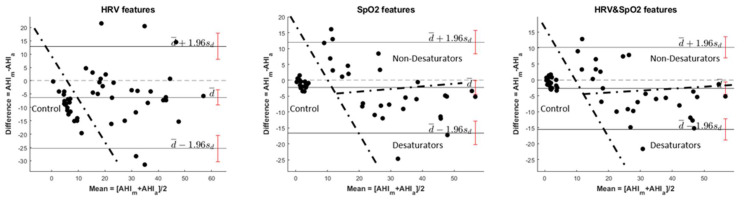
Bland–Altman plots for the automatic (AHIa) versus manual (AHIm) AHI depending on the feature in test patients, with the representation of confidence interval limits for mean and agreement limits.

In Figure 7, we show the Bland–Altman plots for the automatic versus manual AHI in test patients, depending on the features, with the representation of confidence interval limits (95%) for the mean and agreement limits. The bias, standard deviation (Std), and 95% CI limits of Figure 7 are shown in Table 14.

An inspection of the plots let us see some interesting characteristics of the classifiers. In all cases, control patients are grouped in small clusters, and they are well differentiated from desaturating and non-desaturating patients, which are scattered in wider areas. For HRV features, non-control patients are scattered in such a way that it is difficult to find differences among them. On the contrary, for SpO_2_, HRV, and SpO_2_ features, the differences are quite evident. Particularly, AHI for desaturating patients are underestimated, while for non-desaturating, the main tendency is to be overestimated. The confidences around the mean error and agreement limits are quite narrow. We must highlight that, for HRV and SpO_2_ features, we get the narrowest CI.

Regarding the bias, we can see that, in all cases, the value is not zero, thus indicating certain lack of agreement. These biases are not significant because the lines of equality are within the confidence intervals of the mean errors. For SpO_2_, as well as HRV&SpO_2_ features, the biases are very small, and the patients incorrectly classified for AHI limit = 5 are 3. For HRV features, the bias is higher than for the other 2, and a value of −6.2489 compromises the classification of patients more than for the other features (7 patients incorrectly classified).

## 4. Discussion

This is a novel study that thoroughly analyzes the detection of sleep apnea events (quantification) and the diagnosis of OSA patients (per-recording classification), but it focuses on the system performance when the database includes patients who do not always show desaturations during apneic episodes (non-desaturating patients). In this context, this work aims to quantify the system improvement by combining information extracted from different signals (HRV and SpO_2_), mainly, in non-desaturating patients. The HRV features include spectral (Filterbank), cepstral, and nonlinear information (DFA and RQA), whereas SpO_2_ features consider temporal (1 min and 5 min variance) and spectral (Filterbank) information.

The main contributions to the state of the art research are threefold. On the one hand, and for the first time in the literature, we evaluate how non-desaturating patients affect the performance of an automatic apnea detection system. This is particularly important given that, at present, there is no overall accepted alternative system to polysomnography that is simple, automatic, and requires fewer signals. This is due, among other things, to the lack of studies in the literature dealing with OSA patients with other concomitant pathologies. Very few studies have assessed OSA in patients who also suffered from COPD [54,117,118]. The most recent study, carried out by Andrés-Blanco et al. [54], was meant to assess the influence of COPD on an automatic OSA detection system’s performance using the oximeter signal in the patient’s home. For this, they compared the results from polysomnography in the hospital with those obtained at the patient’s home. The study distinguished between OSA patients with and without COPD. However, regarding COPD, it is important to highlight that, within the AASM recommendations [5], OSA analysis with portable systems in patients with COPD is, in principle, not recommended.

On the other hand, it is important to highlight that, unlike other works in the literature that only focus on OSA diagnosis [36,40,41,42,47,49,50,64], we perform real-time detection of apneic events, which allows us to use the system in the context of OSA treatment [53].

Finally, the detection of the most representative features of the underlying system when using HRV or SpO_2_ features alone, or when combined, allows us to deepen the characterization of the physiological system associated with OSA.

### 4.1. Evaluation of HRV and SpO_2_ Signals in Apnea Detection and OSA Diagnosis

The objective of this section is to unify the conclusions reached in Section 3.1 and Section 3.2, which are devoted to per-segment (apneic/non-apneic minute) and per-recording (control patient/desaturating patient/non-desaturating patient) classification, respectively, to define the proposed system.

After analyzing Table 6 (results of minute-by-minute classification in desaturating patients in HuGCDN2014-OXI), we considered that using SpO_2_ variables was the best option to reach high success rates and sensitivities with the lowest number of variables (15). However, given that the subjects under analysis can be both desaturators and non-desaturators, the sensitivities observed in Table 7 (results of minute-by-minute classification in non-desaturating patients in HuGCDN2014-OXI) would lean against exclusively employing SpO_2_ features. Thus, we propose combining HRV and SpO_2_ variables as the best option. This would improve sensitivities in non-desaturating patients significantly. Moreover, the error rates and sensitivities of desaturating patients would not worsen because the results with SpO_2_, as well as HRV and SpO_2_, features are very similar. However, this decision would, to a certain extent, harm non-desaturating patients, as exclusively using HRV variables yields better sensitivities. Nonetheless, desaturating patients are more common in general.

On the other hand, if the system could include only one signal, a method which best harmonizes sensitivities in both desaturators and non-desaturators, then that would be the electrocardiographic signal. In this case, classification rates would be worse. However, using only the oximeter signal would result in sensitivities that were too small in non-desaturating patients.

Concerning the per-recording classification, if only one signal is available, we would also choose the HRV signal and set the AHI limit at 15, thus better balancing sensitivity and specificity (see Figure 6). Using only the SpO_2_ signal would result in sensitivities being too low in non-desaturating patients. However, we must point out that, whenever the system includes features extracted from SpO_2_ (SpO_2_, as well as HRV and SpO_2_), the resulting specificity values are high (see Figure 6). The latter had already been referred to by other authors when evaluating the results from classic oximetric indices (ODIs and CTs). They produce, in general, high specificities but low sensitivities [32]. On the other hand, if including two signals were possible, combining HRV and SpO_2_ signals would be recommended along with using an AHI limit of 5. These conditions would give the best results, which would be close to 100%, for the per-recording classification rate regardless of the type of subject considered (control, desaturator, and non-desaturator) and, especially, the sensitivity in the classification of non-desaturating patients would improve.

In the literature, there are several works that compare the ECG signal with the SpO_2_ signal performance [60], and others analyze the advantages of combining them [57]. However, none differentiate patients who are not desaturators. Xie and Minn [60] concluded that SpO_2_ features obtained better results than ECG features in terms of their diagnostic capability. Based on our results (see Figure 6), we can suggest that this statement is entirely valid for all those patients with a clear desaturation pattern during apneas, but it is not applicable to non-desaturating patients. Regarding the combination of HRV and SpO_2_ variables, these same authors highlighted how convenient information contained in the HRV is a complement to the oximeter information in the OSA diagnosis. Although they focused on the study of the oximetric signal, Hang et al. [52] concluded that including HRV features could boost OSA diagnosis system performance.

Given the recent technological developments in wearable and portable systems, pulse oximeters and ECG monitors that employ Bluetooth [86,119,120,121] are now available, which allow data to be transmitted in real-time to a computer, smartphone, or tablet for analysis. This leads us to consider that a system that includes both signals can be easily and comfortably implemented for the patient, who would undergo the diagnostic test at home via a completely wireless system. In the state of the art research, there are some examples of physiological signal recording systems that send information to a smartphone, highlighting the proposed system’s viability [122].

### 4.2. Selected Features

Of the features selected when the system includes HRV variables exclusively, we can conclude that most of those selected in Physionet are also selected in HuGCDN2014-OXI. Analyzing the features chosen in each case, we can see that combining different characteristics—in this case, cepstral, spectral, and non-linear (RQA and DFA) characteristics—offers the most benefits, as they provide complementary information on the phenomena that occur during apneic events. These results suggest that, in addition to cepstral and frequency information, short-term correlations and recurrences in the signal play an important role in the classification process. In particular, the first cepstrum coefficient and α_1_ (DFA) are selected in both databases, as they are in our previous work [22]. However, by including RQA measures, the spectral information of the HRV becomes less relevant. Regarding the RQA measures, the selection algorithm emphasizes the importance of information on recurrence times (T2) and on the degree of local grouping (Trans and Clust). These measures already stood out in one of our previous studies [87], in which the HRV analysis included exclusively RQA measures.

When the system only considers the SpO_2_ features, the selection (see Table 9 and Table 10) always contains the 1 min and 5 min variance at relevant ranking positions, regardless of the database. Moreover, it also includes many variables corresponding to the spectral information (filter bank outputs). These results differ, to some extent, from those obtained by Ravelo et al. [61], in which the selection algorithm ruled out the 5 min variance. Several studies in the literature show, as does the present work, that basic temporal statistical analyses yield good results with very low computational costs [34,35,45,63,85]. However, some systems that use spectral analysis of oximetric signals obtain good results [30,34,40,45].

Finally, the SpO_2_ features stand out in the selection when combining HRV and SpO_2_ variables. As seen in the previous analysis, the selected set includes the two variances (1 min and 5 min), as in the study carried out by Ravelo et al. [61]. Regarding the HRV variables, the feature selection algorithm rules out cepstrum coefficients and almost all variables related to its spectral content. The DFA variable, α_1_, remains in the HuGCDN2014-OXI database. However, almost all RQA measures, selected when working only with HRV, are included. These results suggest that, once the system includes the oximeter signal, information related to recurrences in the HRV, contained in the RQA measures, plays a very important role in the classification process, unlike the spectral and cepstral information of the HRV. Especially relevant are variables related to the diagonal structures of the RPs (DET), to the vertical structures (LAM and TT), to recurrence times (RT, T2, RTmax, and ENTW), and to the degree of local grouping (Clust and Trans). These features coincide, to a large extent, with those found in one of our previous studies [87], when using the FAN Method: Clust, LAM, RTmax, T1, and DET.

The set of features obtained by combining the HRV and SpO_2_ signals shows how important oximetric variables are compared with those extracted from the HRV, which is consistent with conclusions reached by other authors [60,61]. This is mainly because SpO_2_ is directly linked to the amount of oxygen entering the lungs during inhalation. Hence, an apneic event will result, mostly, in oxygen saturation variation due to airflow interruption. However, the HRV, obtained from the ECG signal, not only reflects the phenomena associated with OSA but can manifest many other concomitant disorders. That being said, according to the arguments presented above, we must highlight the importance of HRV variables, especially in non-desaturating patients.

### 4.3. Desaturation in the Presence of Apneas

In the automatic detection of apneic episodes, especially when based on the SpO_2_ signal, there are several factors that can cause significant differences between AHI and ODI, which are common in patients who we have defined as non-desaturators. These include the following: (1) limitations of pulse oximeters [49], whose accuracy may be conditioned by factors such as skin pigmentation, blood flow in the evaluation area, SpO_2_ values below 80%, artifacts, or disconnections due to patient movement, etc. This is especially important in OSA-diagnosed patients, whose peripheral blood perfusion is often limited; (2) hypopneas, which imply a decrease in airflow, are factored into AHI calculations. The main problem is that marked desaturation is not always present during a hypopnea. In this regard, we must point out that neither of the two databases used for the experiments distinguish between apneas and hypopneas; (3) short duration apneas that do not cause desaturations; (4) upper airway resistance syndrome, in which the patient does not show apneas or desaturations but, rather, repetitive arousals due to a progressive increase in intrapleural pressure, increased work of breathing, and daytime drowsiness. In the past, this disorder was not considered sleep apnea; however, the latest AASM recommendations suggest including it within this group of pathology [55,123]; (5) displacements of the hemoglobin dissociation curve due to a variation in partial pressure of carbon dioxide (pCO_2_), temperature, pH, or 2–3 diphosphoglycerate (2–3 DPG), among others. Repeated apneic events usually lead to recurrent hypoxias (SpO_2_ values lower than 90%) and hypercapnias (abnormal increase in carbon dioxide (CO_2_)) in arterial blood. The pH decreases as the concentration of CO_2_ increases. Moreover, hypoxias lead to a rise in 2–3 DPG, which binds to hemoglobin, thus reducing hemoglobin oxygen affinity. It is also important to note that how these factors affect the curve displacement is specific to each person. This would explain the different responses observed in patients due to airway obstructions of the same duration. All these circumstances contribute to a rightward shift of the hemoglobin dissociation curve, a sign of hemoglobin’s decreased affinity for oxygen that favors the release of oxygen in the tissues.

On the other hand, there are also changes in the structure and amount of blood hemoglobin that can lead to particularly high (such as in methemoglobinemia or carboxyhemoglobinemia) or low (such as in anemia) oximetry readings that are not related to respiratory disturbances [55]. Another relevant factor is obesity, as oximeter data in this type of patient are not reliable for OSA diagnosis [55,124].

Last, a final observation on chemoreceptors and baroreceptors in OSA patients. Carlson et al. [125] found out that OSA patients show arterial baroreceptor reflex attenuation or inhibition, due to chemoreceptors stimulation. In the same context, Narkiewicz et al. [126] studied chemoreceptor sensitivity and set out that it is increased in OSA patients. Desaturation, accompanied by hypercapnia, stimulates ventilation because of chemoreceptors, so rapid increases in ventilation lead to resaturation and a decrease in the duration of apneas and hypopneas.

All these factors reinforce how important it is to avoid basing OSA diagnosis exclusively on the SpO_2_ signal. Furthermore, with this work, we open the door to the possibility that, in the future, respiratory events can be studied thoroughly by attending, for example, to their duration. It is possible that the effects on sleep quality and, in general, on patients’ quality of life depend on the duration of the respiratory events since, in short ones, desaturations may not occur.

### 4.4. Limitations of the Proposed Method

Despite its contributions, we must draw attention to some limitations present in the study. To a large extent, these limitations are related to the databases employed. In the widely used Physionet database, there are only eight patients with all the signals involved in this analysis—ECG and SpO_2_. Including them allows us to compare our results with other works in the literature, although conclusions drawn from such a small database are limited. In any case, it is necessary to bear in mind that, as our main objective is to evaluate the system considering non-desaturating patients, our most important conclusions came from the HuGCDN2014-OXI database, whose main limitation is that it only accounts for control subjects and patients with severe OSA. We should also mention that neither database includes subjects with significant concomitant disorders, such as cardiorespiratory diseases. Additional studies with this type of patient would be necessary to give our conclusions a more generalizable character. All these circumstances are especially relevant considering that HRV, in the context of apnea, may be conditioned by the integrity of sympathetic and parasympathetic stimuli, so associated pathologies, such as diabetes mellitus or chronic heart failure, could cause a limited response of the autonomic system and, therefore, a decrease in HRV [127,128,129]. Moreover, there are also some studies suggesting that the autonomic nervous system activity is age-dependent, which could lead to a decrease in HRV in healthy subjects [127,130,131,132]. The latter led Zamarrón et al. to think that combining HRV and SpO_2_ signals was especially important in elderly patients. On the other hand, Gutiérrez-Tobal et al. [27] studied the potential gender differences in HRV sleep apnea information. The conclusions of these works could suggest that assessing automatic systems for OSA diagnosis should differentiate pathologies, age, and sex of the subjects under study.

We must bear in mind that most state of the art research aimed at diagnosing OSA or apneic events from HRV uses public databases. However, those that also include oximetry usually rely on their own databases, making it difficult to compare the different systems. In our opinion, more efforts should be made to create a database to replace the widely used Physionet. The new database should include ECG signals, SpO_2_ signals, and a sufficiently high number of subjects to allow the study of special interest groups. In this sense, it would be useful to include a greater range of ages, a greater number of women, subjects of different races, and patients with different diseases that may affect OSA diagnosis, especially pulmonary and cardiac diseases. This would also allow results from different systems to be compared. As a result, widely contrasted solutions could be reached to create a universal automatic system that is acceptable to the medical community.

Finally, we must point out that our proposal is intended to be a portable or wearable system. However, the signals used in the analysis were collected in a controlled hospital environment. Therefore, we should evaluate its performance in an unsupervised environment to validate the conclusions obtained.

### 4.5. Comparison with Prior Work

As already discussed in Section 1.2, in the state of the art research, there are many studies that use the oximeter signal or the combination of HRV and SpO_2_ signals for OSA diagnosis. The problem when comparing our results with those reached in previous studies is the use, in most cases, of diverse private databases: the proportion of control subjects versus patients in the different OSA grades, concomitant diseases, desaturating characteristics, age, and physical attributes of the subjects under study, etc. These aspects are particularly relevant as obesity or the presence of COPD can increase the number of false positives [80].

However, general conclusions can be drawn depending on the type of features used in each case. The classical oximetric indices (delta index, ODIs, and CTs) show certain limitations that have been exposed in previous studies [36,45]. Of such limitations, one is records analysis, which can only be carried out offline. This allows overall classification of the subjects as pathological or non-pathological, but it is impossible to analyze apneic events in real time. Several authors have reported difficulty in performing an overall analysis in real time, i.e., obtaining screening results in a few minutes. Fortunately, the computational power of modern servers allows this task to be approached without appreciable delay. As mentioned in 4.1, recent technological developments in wearable and portable systems are now available, which allow data to be transmitted in real-time to a computer, smartphone or tablet for analysis. Additionally, there are examples of physiological signal recording systems that send information to a smartphone, highlighting the proposed system’s viability. The sensitivities and specificities obtained vary greatly between the different studies (sensitivities between 30% and 98%, and specificities between 41% and 100% [50]), and the correlation between AHI and the oximetric indices is low [46]. Moreover, the results are generally worse than those obtained when other features are introduced, including temporal and frequency statistics, spectral analysis, and nonlinear analysis [42,45,48,85]. For meaningful comparison, we focus on the studies that use Physionet, especially those that include only the eight patients with both signals (ECG and SpO_2_). Table 15 shows the results obtained for per-segment classification because the small number of patients does not allow, in general, an adequate analysis of per-recording classification. Lee et al. [37] introduced the wavelet transform with an accuracy of 96.55%, sensitivity of 95.74%, and specificity of 97.02% by defining a threshold for each patient’s own wavelet coefficients, which is not feasible in clinical practice. Therefore, the reference values for this study would be those shown in the second row and obtained with a global threshold (Acc: 82.70%; Sens: 78.99%; Spe: 84.82%). Burgos et al. [43] used a modified version of the classical oximetric indices to adapt them to a segmental study (Acc: 93.03%; Sens: 92.35%; Spe: 93.52%). The two authors referred to so far only made use of the SpO_2_ signal. Shi et al. [59] performed a comparative study dependent on the type of signal used, extracting time and frequency features of each signal. Wang et al. [133] used a residual network with HRV that was 94.39% accurate. With a self-configuring classifier combination, Mostafa et al. [134] reached an accuracy, sensitivity and specificity of 91.33%, 98.11%, and 86.98%. Mostafa et al. [83] reached an accuracy, sensitivity, and specificity of 94.24%, 92.04%, and 95.78% using a convolutional neural network with multi-objective hyperparameter optimization. In another study, Mostafa et al. [82] reached an accuracy, sensitivity, and specificity of 95.14%, 92.36%, and 97.08%, respectively, with a greedy based convolutional Neural Network. Bernardini et al. [135] proposed a convolutional deep learning architecture to reduce the temporal resolution of raw waveform data and reached an accuracy, sensitivity, and specificity of 93.60%, 91.20%, and 95.10%. Finally, Sharma et al. [136] decomposed the SpO_2_ signals into various sub-bands (SBs) and extracted Shannon entropy features (Acc.: 95.97%; Sens.: 95.78% and Spe.: 96.09%).

Comparing the results from our study for the Physionet database with those reached in other works, we can conclude that, both for the cases in which the HRV and SpO_2_ signals are used individually and those where they are used jointly, our proposal shows interesting and competitive results that emphasize the physical information obtained from the features, along with a simpler classification method.

Although the HuGCDN2014-OXI database is not exactly the same as the one used by Ravelo-García et al. [61], Mostafa et al. [82,83,134], and Mendoça et al. [137], the data were collected in the same sleep laboratory, which is why we consider it appropriate to compare the results obtained with both datasets in Table 16. Ravelo et al. [61] obtained the following results by combining SpO_2_ and ECG (Acc: 86.9%; Sens: 73.4%; Spe: 92.3%). Mostafa et al. [134] reached an accuracy, sensitivity and specificity of 85.30%, 82.48% and 86.28% respectively. Mendonça et al. [137] obtained an accuracy, sensitivity, specificity, and AUC of 88%, 80%, 91%, and 0.86, respectively. Mostafa et al. [83] reached an accuracy, sensitivity, and specificity of 89.32%, 74.75%, and 94.44%, respectively. Finally, Mostafa et al. [82] reached an accuracy, sensitivity and specificity of 88.49%, 73.64%, and 93.80%, respectively. In our proposal, sensitivities are notably better and well-balanced with specificities. In return, we obtained lower specificities, all of which are greater than 76.90%. Bearing in mind that this is a medical diagnostic system, we consider the increased sensitivity especially relevant, especially as we also include a high proportion of non-desaturating patients, which further complicates the per-segment classification.

In summary, we consider our results very promising. Nevertheless, there is still margin to improve the global system performance, e.g., increasing the number of features, specifically by adding new non-linear characteristics to both signals that will allow us to obtain additional complementary information; applying other pattern recognition methods as those based on deep learning [138], ANN, k-NN, SVM or decision tree classifiers; assessing other feature selection techniques.

## 5. Conclusions

To the best of our knowledge, this is the first study in the context of OSA that is focused on quantitative analysis of results from patients under study who do not show a clear desaturating pattern during apneic events. For this purpose, we have evaluated the use of features extracted from the HRV and SpO_2_ signals, individually and jointly, in different sets of patients according to their desaturating patterns.

We concluded that the best option, both for the detection of apneic events and for the global diagnosis of OSA patients, is to combine both signals, especially when the subjects under study include patients with a non-desaturating pattern. In that case, we found, for the detection of apneic events in Physionet, a success rate of 96.19%, sensitivity of 95.74%, and specificity of 95.25% (AUC: 0.99) and in HuGCDN2014-OXI, we found rates of 87.32%, 83.81%, and 88.55% (AUC: 0.934), respectively. The results for the global diagnosis of OSA patients (HuGCDN2014-OXI) were: success rate of 95.74%, sensitivity of 100%, and specificity of 89.47%. The AHI limit, in this case, would be set at 5. However, if only one signal is available, we would suggest using the HRV, as otherwise, the sensitivity for non-desaturating patients would be very low. For this option, we found the following results in Physionet: a 92.71% success rate, 92.38% sensitivity, and 93.3% specificity (AUC: 0.983); in HuGCDN2014-OXI: 77.22%, 78.13%, and 76.9% (AUC: 0.854), respectively. For this case, we suggest 15 as an AHI limit.

Regarding the features, we can highlight two cases. If both HRV and SpO_2_ signals are included, results suggest that we should include, of the SpO_2_ signal, both 1 min and 5 min variances, some variables related to the spectral information, and, of the HRV, the RQA measures and the α_1_ variable (DFA). However, if we only used the HRV signal, it would be useful to add cepstrum coefficients and spectral information.

In summary, we can conclude that the proposed system diagnoses OSA well, especially when using both HRV and SpO_2_ signals. Given recent technological breakthroughs in portable and wearable systems, both signals could be recorded wirelessly in patients’ homes, thus avoiding the drawbacks of using polysomnography. Moreover, real-time detection of apneic events would allow for improved treatment, as steps could be taken during apneic episodes to restore normal breathing [51]. Given the important consequences of OSA for patients’ long-term health, this would reduce mortality rates associated with this pathology.

## Figures and Tables

**Table 1:** Relevant studies related to apnea detection using the single lead ECG.

Works	Description
Varon et al. [17]	There are two different features: one describing changes in the morphology of the ECG and one that computes the information shared between respiration and heart rate using orthogonal subspace projection.
Ravelo-García et al. [18]	Symbolic dynamics variables in sleep apnea screening.
Penzel et al. [19]	Comparison of the ability of spectral analysis and detrended fluctuation analysis (DFA) to identify the CVHR in sleep apnea.
Karandikar et al. [25]	RQA applied to HRV and to ECG Derived Respiratory (EDR) signals and different combinations to assess the classification system.
Gutiérrez et al. [27]	Evaluation of spectral entropy (SE) and multiscale entropy (MsE) of HRV signals in sleep apnea, assessing gender differences.
Le et al. [39]	Combination of RQA features and power spectral density (PSD), obtained from the RR series, and Support Vector Machines (SVM) to determine sleep apnea events.
Mendez et al. [70]	Extraction of time and spectral parameters from RR series and R-peak areas by using a time-varying autoregressive model.
Schrader et a. [71]	Analysis of the spectral components of HRV using Fourier and Wavelet Transformation with appropriate application of the Hilbert Transform.
Al-Angari et al. [72]	Nonlinear sample entropy to assess the signal complexity of HRV.
Maier and Dickhaus [74]	The first authors who introduced RQA in sleep apnea studies and compared the results obtained when using recurrence analysis and spectral techniques to HRV.
Mendez et al. [75]	Comparison of system’s performance when sleep apnea is detected using empirical mode decomposition (EMD) vs. wavelet analysis (WA).
Sharma et al. [76]	Hermite basis functions to develop a sleep apnea detection technique using the ECG.
Cheng et al. [77]	Modified version of RQA, heterogeneous RQA (HRQA), applied to sleep apnea.

**Table 2:** Demographic information of Physionet database.

Record	Sex	Age (Years)	Minutes	Nonapnea	Apnea	AHI (Events/h)	BMI (kg/m^2^)
a01	M	51	490	20	470	69.6	33.31
a02	M	38	529	109	420	69.5	37.04
a03	M	54	520	274	246	39.1	28.34
a04	M	52	493	40	453	77.4	40.43
b01	F	44	488	469	19	0.24	21.80
c01	M	31	485	485	0	0	21.86
c02	M	37	503	502	1	0	25.62
c03	M	39	455	455	0	0	19.20

Sex: M (male)/F (female), AHI: apnea-hypopnea index, BMI: body mass index.

**Table 3:** Demographic information of HuGCDN2014-OXI database. Patients are defined as non-desaturating when their ODI is less than half their AHI.

Parameter	Control	Desaturating	Non-Desaturating	All
Sample size (male/female)	38 (28/10)	34 (27/7)	11 (7/4)	83 (62/21)
Age (years)	43.55 ± 12.71	53.97 ± 9.5	49.55 ± 6.68	48.61 ± 11.77
BMI (kg/m^2^)	29.24 ± 6.31	34.36 ± 5.62	30.57 ± 5.98	31.52 ± 6.5
Number of arousals	73.34 ± 28.14	248.85 ± 77.77	224.82 ± 95.59	165.31 ± 105.65
AHI (events/h)	2.04 ± 1.35	57.78 ± 21.08	42.51 ± 18.80	30.24 ± 30.44
ODI (events/h)	0.84 ± 0.74	52.07 ± 20.54	11.91 ± 9.85	23.29 ± 27.89

BMI: body mass index, AHI: apnea-hypopnea index, ODI: oxygen desaturation index.

**Table 4:** RQA features included in the feature set.

Based on Recurrence Density:	Recurrence Rate (REC)
Based on diagonal line structures:	Determinism (DET) Average diagonal line length (L) Length of the longest diagonal line (Lmax) Shannon Entropy (ENTR)
Based on vertical line structures:	Laminarity (LAM) Trapping Time (TT) Maximal length of vertical lines (Vmax)
Recurrence times:	Recurrence Time type 1 (T1) Recurrence Time type 2 (T2) Mean Recurrence Time (RT) Maximal Recurrence Time (RTmax) Minimal Recurrence Frequency (RF) Entropy of White Vertical Lines (ENTW) Recurrence Period Density Entropy (RPDE)
Measures originating in the complex network theory:	Clustering Coefficient (Clust) Transitivity (Trans)

**Table 5:** Per-segment performance (test results) in Physionet, depending on the features. Combining electrocardiographic and oximetric characteristics yields the best results with 12 variables.

Features	N(OR.) ^1^	N(RED.)	ACC	SENS	SPEC	AUC
HRV	73	7	92.71	92.38	93.3	0.983
SpO_2_	22	6	95.76	95.37	94.51	0.986
HRV + SpO_2_	95	12	96.19	95.74	95.25	0.990

^1^ (N(OR.): original number of features, N(RED.): number of selected features, ACC: classification rate (accuracy), SENS: sensitivity, SPEC: specificity, AUC: area under the ROC curve).

**Table 6:** Per-segment performance (test results) in HuGCDN2014-OXI (only desaturating patients), depending on the features. When we consider oximetric variables or combine both types, the results are very similar, reaching values that are approximately 10% higher than those reached only with HRV.

Features	N(OR.) ^1^	N(RED.)	ACC	SENS	SPEC	AUC
HRV	73	17	74.639	82.846	65.888	0.838
SpO_2_	22	15	82.230	98.507	64.871	0.925
HRV + SpO_2_	95	24	82.689	97.967	66.396	0.930

^1^ (N(OR.): original number of features, N(RED.): number of selected features, ACC: classification rate (accuracy), SENS: sensitivity, SPEC: specificity, AUC: area under the ROC curve).

**Table 7:** Per-segment performance (test results) in HuGCDN2014-OXI (only non-desaturating patients), depending on the features. According to classification rates, sensitivities, and AUCs, the values are lower than those reached in desaturating patients.

Features	N(OR.) ^1^	N(RED.)	ACC	SENS	SPEC	AUC
HRV	73	17	72.230	72.278	72.205	0.777
SpO_2_	22	15	76.374	51.236	89.481	0.829
HRV + SpO_2_	95	24	77.816	59.786	87.217	0.847

^1^ (N(OR.): original number of features, N(RED.): number of selected features, ACC: classification rate (accuracy), SENS: sensitivity, SPEC: specificity, AUC: area under the ROC curve).

**Table 8:** Per-Segment performance (test results) in HUGCDN2014-OXI (control subjects, desaturating, and non-desaturating patients), depending on the features. These results represent a real system, tested with healthy subjects and OSA diagnosed patients (desaturating and non-desaturating). For the main parameters (classification rate, sensitivity, and AUC), using only the HRV features yields the worst results, which improve using only SpO_2_ variables, but we obtain the best results by combining both feature types.

Features	N(OR.) ^1^	N(RED.)	ACC	SENS	SPEC	AUC
HRV	73	17	77.220	78.127	76.904	0.854
SpO_2_	22	15	86.782	81.683	88.564	0.926
HRV + SpO_2_	95	24	87.323	83.812	88.549	0.934

^1^ (N(OR.): original number of features, N(RED.): number of selected features, ACC: classification rate (accuracy), SENS: sensitivity, SPEC: specificity, AUC: area under the ROC curve).

**Table 9:** Selected features after the feature selection process in Physionet. The order in which they appear is in agreement with the feature ranking created according to the number of times a certain feature is selected by the sequential forward feature selection method. The three columns contain the chosen variables when HRV, SpO_2_, or HRV + SpO_2_ features are used.

HRV (7 → 6)	SpO_2_ (6)	HRV + SpO_2_ (12 → 7)
CC1	VarSAT_5m_	VarSAT_5m_
CC8	VarSAT_1m_	VarSAT_1m_
T2	FbSAT 17	FbSAT 1
CC4	FbSAT 1	T2
FbHRV 21	FbSAT 4	FbSAT 13
α_1_	FbSAT 12	FbSAT 16
Trans		FbSAT 17
		Clust
		TT
		RT
		RTmax
		FbSAT 12

**Table 10:** Selected features after the feature selection process in HUGCDN2014-OXI. The order in which they appear is in agreement with the feature ranking created according to the number of times a certain feature is selected by the sequential forward feature selection method. The three columns contain the chosen variables when HRV, SpO_2_, or HRV + SpO_2_ features are used.

HRV (17)	SpO_2_ (15 → 9)	HRV + SpO_2_ (24 → 17)
α_1_	varSAT1m	varSAT1m
CC1	FbSAT 1	Clust
RT	FbSAT 10	FbSAT 1
FbHRV 2	FbSAT 20	a _1_
Clust	FbSAT 9	Trans
T2	FbSAT 2	varSAT5m
α_2_	FbSAT 17	FbSAT 10
L	FbSAT 5	FbSAT 20
DET	varSAT5m	DET
CC4	FbSAT 19	FbSAT 9
FbHRV 11	FbSAT 12	FbSAT 5
LAM	FbSAT 13	T2
Trans	FbSAT 7	FbSAT 2
CC17	FbSAT 15	RT
RTmax	FbSAT 4	FbHRV 1
FbHRV 1		LAM
FbHRV 15		FbSAT 17
		FbSAT 12
		TT
		Lmax
		FbHRV 30
		ENTW
		FbHRV 23
		FbSAT 7

**Table 11:** Statistical description (median and interquartile ranges) of the features according to the presence or absence of apneic minutes in the Physionet database. We applied the Wilcoxon test to compare statistical significance in the two groups under study. The test was performed using a level of significance *p* = 0.05, and values of *p* < 0.05 were considered significant.

Features	Median (Non−Apneic)	Median (Apneic)	*p*
FbHRV 21	−5.66 (−6.35; −4.99)	−6.06 (−6.57; −5.61)	<0.0001
CC 1	−0.90 (−1.52; −0.45)	−0.55 (−1.30; −0.26)	<0.001
CC 4	−0.01 (−0.05; 0.04)	0.14 (0.08; 0.19)	<0.0001
CC 8	−0.07 (−0.11; −0.02)	−0.04 (−0.07; −0.02)	<0.0001
α_1_	1.04 (0.83; 1.31)	1.73 (1.55; 1.83)	<0.0001
TT	2.35 (2.16; 2.76)	2.90 (2.64; 3.27)	<0.0001
T2	23.34 (21.35; 25.96)	34.54 (31.52; 37.81)	<0.0001
Trans	0.26 (0.23; 0.30)	0.40 (0.37; 0.43)	<0.0001
RT	23.21 (20.86; 26.74)	37.71 (33.71; 42.30)	<0.0001
RTmax	174 (143; 213)	186 (154; 230)	<0.001
Clust	0.28 (0.25; 0.32)	0.43 (0.40; 0.46)	<0.0001
FbSAT 1	−8.8⋅10^−3^ (−1.21⋅10^−2^; −5.9⋅10^−3^)	−9.48⋅10^−4^ (−1.2⋅10^−3^; −7.31⋅10^−4^)	<0.0001
FbSAT 2	−5.38 (−5.78; −5.06)	−7.72 (−7.99; −7.45)	<0.0001
FbSAT 4	−7.17 (−7.55; −6.87)	−9.16 (−9.41; −8.91)	<0.0001
FbSAT 12	−9.46 (−9.86; −9.16)	−11.38 (−11.65; −11.16)	<0.0001
FbSAT 13	−9.60 (−9.99; −9.28)	−11.63 (−11.89; −11.39)	<0.0001
FbSAT 16	−9.97 (−10.37; −9.66)	−12.21 (−12.52; −11.93)	<0.0001
FbSAT 17	−10.48 (−10.85; −10.16)	−12.00 (−12.23; −11.79)	<0.0001
varSAT_1m_	0.21 (0.13; 0.39)	3.58 (3.08; 3.97)	<0.0001
varSAT_5m_	0.28 (0.20; 0.48)	3.71 (3.41; 4.03)	<0.0001

**Table 12:** Statistical description (median and interquartile ranges) of the features according to the presence or absence of apneic minutes in the HUGCDN2014-OXI database. We applied the Wilcoxon test to compare statistical significance in the two groups under study. The test was performed using a level of significance *p* = 0.05, and values of *p* < 0.05 were considered significant.

Features	Median (Non−Apneic)	Median (Apneic)	*p*
FbHRV 1	−1.53 (−2.27; −0.92)	−1.77 (−2.33; −1.19)	<0.05
FbHRV 2	−1.93 (−2.42; −1.52)	−1.36 (−1.75; −1.06)	<0.0001
FbHRV 11	−5.08 (−5.72; −4.45)	−5.30 (−5.86; −4.80)	<0.01
FbHRV 15	−5.26 (−6.05; −4.32)	−5.59 (−6.31; −4.78)	<0.001
FbHRV 23	−5.90 (−6.72; −4.96)	−6.04 (−6.73; −5.32)	NS
FbHRV 30	−6.43 (−7.19; −5.59)	−6.60 (−7.34; −5.74)	NS
CC 1	−1.36 (−1.78; −0.98)	−1.06 (−1.52; −0.80)	<0.0001
CC 4	0.06 (−0.02; 0.15)	0.10 (0.02; 0.18)	<0.001
CC 17	−0.06 (−0.08; −0.03)	−0.07 (−0.09; −0.04)	<0.05
α_1_	1.17 (0.94; 1.39)	1.49 (1.32; 1.64)	<0.0001
α_2_	0.72 (0.50; 0.98)	0.53 (0.36; 0.77)	<0.0001
DET	0.53 (0.37; 0.67)	0.64 (0.54; 0.75)	<0.0001
L	3.08 (2.56; 3.75)	3.55 (3.03; 4.50)	<0.0001
Lmax	58 (24; 117)	91 (53; 163)	<0.0001
LAM	0.53 (0.31; 0.69)	0.69 (0.58; 0.78)	<0.0001
TT	2.54 (2.21; 2.92)	2.82 (2.61; 3.13)	<0.0001
T2	22.55 (19.41; 26.64)	28.93 (24.50; 33.27)	<0.0001
Trans	0.27 (0.23; 0.31)	0.33 (0.28; 0.38)	<0.0001
RT	22.88 (18.91; 28.78)	31.37 (25.84; 37.79)	<0.0001
RTmax	188 (156; 224)	197 (169; 227)	<0.001
ENTW	3.52 (3.26; 3.76)	3.61 (3.39; 3.82)	<0.001
Clust	0.29 (0.25; 0.34)	0.36 (0.30; 0.41)	<0.0001
FbSAT 1	−5.8⋅10^−3^ (−8⋅10^−3^; −4⋅10^−3^)	−5.6⋅10^−3^ (−7.1⋅10^−3^; −4.4⋅10^−3^)	NS
FbSAT 2	−5.77 (−6.14; −5.44)	−5.82 (−6.07; −5.57)	<0.05
FbSAT 4	−7.60 (−7.95;−7.28	−7.59 (−7.84; −7.36)	NS
FbSAT 5	−8.12 (−8.48; −7.81)	−8.13 (−8.38; −7.90)	NS
FbSAT 7	−8.87 (−9.24; −8.55)	−8.91 (−9.16; −8.67)	NS
FbSAT 9	−9.46 (−9.81; −9.15)	−9.48 (−9.71; −9.24)	NS
FbSAT 10	−9.69 (−10.05; −9.37)	−9.72 (−9.97; −9.48)	NS
FbSAT 12	−10.11 (−10.47; −9.80)	−10.11 (−10.36; −9.87)	NS
FbSAT 13	−10.31 (−10.67; −10.01)	−10.32 (−10.55; −10.09)	NS
FbSAT 15	−10.59 (−10.96; −10.27)	−10.63 (−10.88; −10.38)	NS
FbSAT 17	−10.83 (−11.20; −10.49)	−10.83 (−11.09; −10.59)	NS
FbSAT 19	−10.95 (−11.34; −10.60)	−10.99 (−11.27; −10.74)	NS
FbSAT 20	−10.99 (−11.39; −10.64)	−11.02 (−11.30; −10.76)	NS
varSAT1m	0.20 (0.08; 0.39)	1.97 (1.28; 2.87)	<0.0001
varSAT5m	0.30 (0.18; 0.62)	2.13 (1.47; 2.93)	<0.0001

**Table 13:** Per-recording performance (test results) in HUGCDN2014-OXI (control subjects, desaturating, and non-desaturating patients), depending on the features and on the AHI limit. These results represent a real system, tested with healthy subjects and OSA diagnosed patients (desaturating and non-desaturating). For the main parameters (classification rate and sensitivity), we obtain the best results by combining both feature types (highlighted in gray).

Features	AHI Limit	ACC	SENS	SPEC
HRV	5	63.83	100	10.53
	10	76.60	96.43	47.37
	15	85.11	89.29	78.95
SpO_2_	5	95.74	92.86	100
	10	91.49	85.71	100
	15	85.11	75	100
HRV and SpO_2_	5	95.74	100	89.47
	10	93.62	89.29	100
	15	87.23	78.57	100

ACC: classification rate (accuracy), SENS: sensitivity, SPEC: specificity.

**Table 14:** Bias, standard deviation (Std), and 95% CI limits of Figure 7.

Features	Bias	Std	CI
HRV	−6.2489	9.7682	[−25.3942, 12.8965]
SpO_2_	−2.2865	7.3125	[−16.6188, 12.0458]
HRV & SpO_2_	−2.6295	6.5822	[−15.5304, 10.2714]

**Table 15:** Comparison of the per-segment classification results obtained in Physionet.

Works	Year	Signals	AUC	Acc (%)	Sens (%)	Spe (%)
[37]	2004	SpO_2_		96.55 82.70	95.74 78.99	97.02 84.82
[43]	2010	SpO_2_	0.985	93.03	92.35	93.52
[59]	2011	SpO_2_ ECG		94 89.97	94 87.69	94 91.18
[133]	2019	ECG		94.39	93.04	94.95
[134]	2019	SpO_2_		91.33	98.11	86.98
[83]	2020	SpO_2_		92.24	92.04	95.78
[82]	2020	SpO_2_		95.14	92.36	97.08
[135]	2021	ECG		93.60	91.20	95.10
[136]	2022	SpO_2_	0.98	95.97	95.78	96.09
Our proposal		SpO_2_	0.986	95.76	95.37	94.51
		ECG	0.983	92.71	92.38	93.3
		SpO_2_ + ECG	0.990	96.19	95.74	95.25

**Table 16:** Comparison of the per-segment classification results obtained in two databases collected in the Hospital Universitario de Gran Canaria Dr. Negrín.

Works	Dataset	Year	Signals	AUC	Acc (%)	Sens (%)	Spe (%)
[61]	HuGCDN2008	2015	SpO_2_ ECG SpO_2_ + ECG	0.898 0.809 0.919	86.5 79.4 86.9	75.6 42.4 73.4	91 94.3 92.3
[134]	HuGCDN2008	2019	SpO_2_	-	85.3	82.48	86.28
[137]	HuGCDN2008	2020	SpO_2_	0.86	88	80	91
[83]	HuGCDN2008	2020	SpO_2_		89.32	74.75	94.44
[82]	HuGCDN2008	2020	SpO_2_		88.49	73.64	93.80
Our proposal	HuGCDN2014-OXI		SpO_2_ ECG SpO_2_ + ECG	0.926 0.854 0.934	86.78 77.22 87.32	81.68 78.13 83.81	88.56 76.90 88.55

## Data Availability

The data presented in this study (HuGCDN2014-OXI database) are openly available in Mendeley at https://data.mendeley.com/datasets/cdxs63gdzc/1 accessed on 17 April 2023.
